# Maximal Shannon entropy in the vicinity of an exceptional point in an open microcavity

**DOI:** 10.1038/s41598-020-69479-w

**Published:** 2020-07-28

**Authors:** Kyu-Won Park, Jinuk Kim, Songky Moon, Kyungwon An

**Affiliations:** 1 Department of Physics and Astronomy & Institute of Applied Physics, Seoul National University, Seoul, 08826 Korea; 2 Faculty of Liberal Education, Seoul National University, Seoul, 08826 Korea

**Keywords:** Quantum mechanics, Information theory and computation, Quantum optics, Micro-optics

## Abstract

The Shannon entropy as a measure of information contents is investigated around an exceptional point (EP) in an open elliptical microcavity as a non-Hermitian system. The Shannon entropy is maximized near the EP in the parameter space for two interacting modes, but the exact maximum position is slightly off the EP toward the weak interaction region while the slopes of the Shannon entropies diverge at the EP. The Shannon entropies also show discontinuity across a specific line in the parameter space, directly related to the exchange of the Shannon entropy as well as the mode patterns with that line as a boundary. This feature results in a nontrivial topological structure of the Shannon entropy surfaces.

## Introduction

Any real physical system is open since it inevitably interacts with its surroundings. To investigate such a system, it is convenient to introduce a non-Hermitian Hamiltonian based on the system-bath interaction model^1^. In a non-Hermitian system, the openness effects are dramatically exhibited in the vicinity of a singular point where two interacting modes coalesce, i.e., their eigenvalues and eigenvectors coincide, respectively^2^. This singular point, never occurring in a closed system, is called an exceptional point (EP).

EP has recently been extensively studied in various systems such as cold atoms^3^, carbon nanotubes^4^, nanowires^5^, photonic crystal slabs^6^, electrical circuit resonators^7^, optical microcavities^8^, magnon-polariton systems^9^, ultrasonic acoustic cavities^10^, and so on, both theoretically and experimentally. They have not only led to useful applications such as microcavity sensors^11,12^ and time asymmetric loop for optical communication band^13^ but also revealed many intriguing phenomena related to parity-time symmetry^14–16^, chirality^17,18^, phase transition^19,20^, mode switching^21^ and topological transfer of energy^22^.

One of the most intriguing effects associated with EP is the radiative properties of atoms interacting with two resonance modes in an open cavity. The atomic spontaneous emission in an open cavity is enhanced by the Petermann factor^23^ due to non-orthogonality of modes. Such enhancement has been experimentally confirmed in various devices^24–26^. It is also predicted that the Petermann factor diverges at an EP^27^, which implies the atomic spontaneous emission would be greatly enhanced under this condition. The Petermann factor is also interpreted as excess noise^28^, suggesting the linewidth of a laser operating at an EP would be greatly broadened beyond the Schawlow-Townes limit.

Recently, there has been a report of experimentally observing increased linewidth of phonon lasing coupled to two cavity modes coalescing at an EP, supporting this line of reasoning^29^. However, there is a conflicting theory suggesting negligible enhancement in spontaneous emission at an EP due to coherent perfect cancellation of two diverging Petermann factors associated with two cavity modes^30^; the theory is classical in parts, and only the bi-orthogonality of the cavity modes is taken into account whereas the spatial distributions of the cavity modes are neglected. The reason why this conflict arises and which interpretation of the spontaneous emission near EP is valid are still open questions.

It is worth noting that at an EP the spatial integral of the square of the mode function over the cavity completely vanishes^27^, which implies the mode function itself would become spatially irregular and disordered in both real and imaginary parts. It is therefore natural to conjecture that the enhancement in spontaneous emission as well as the excess noise associated with the diverging Petermann factor is to some degree related to this disordered mode pattern. One is thus led to ask how the information contents associated with the mode distribution behave near an EP in an open physical system in term of the Shannon entropy, which as a measure of average information contents is directly related to the degree of the irregular^31^ or disorder physical quantities^32^.

In this paper, we take on this intriguing question by introducing the Shannon entropy for the probability density of eigenmodes and applying it around an EP in a dielectric microcavity. The Shannon entropy is originally defined as a measure of average information contents associated with random outcomes in data communication^33^ and information theory^34^. It has been utilized in diverse research areas to quantify amount of information, including black holes^35^, confined hydrogen-like systems^36^ and quantum entanglement^37^. The Shannon entropy has also been used as an indicator for avoided crossing in dielectric microcavities^38^ and related to the quantum transition from order to chaos^39^.

### Eigenmodes and eigenvalues in an elliptical dielectric microcavity

Before we evaluate Shannon entropy, let us first discuss the physical system that we consider. Assume a closed physical system described by a Hermitian Hamiltonian ${H}_{\mathrm{S}}$. Let us suppose it is allowed to interact with a bath and thus becomes an open system. The resulting open system can be described by a non-Hermitian Hamiltonian formulated by

1 $$\begin{aligned} H=H_{\mathrm{S}}+V_{{\mathrm{SB}}}G_{\mathrm{B}}^{(\mathrm{out})}V_{\mathrm{BS}}, \end{aligned}$$

where ${G}_{\mathrm{B}}^{(\mathrm{out})}$ is an outgoing Green function in a bath, and ${V}_{\mathrm{SB}}$($V_{\mathrm{BS}}$) is the interaction from the bath (the closed system) to the closed system (the bath)^1^. It should be noted that the domain of *H* and thus that of its eigenvectors is restricted to the part of the system excluding the bath^1,40^. If we pay attention to two particular eigenstates interacting with each other, with the interaction with the other states negligible, the non-Hermitian Hamiltonian can modeled in a 2-by-2 matrix form as

2 $$\begin{aligned} H= \begin{pmatrix} \epsilon _{1} &{} g \\ g &{} \epsilon _{2} \end{pmatrix}, \end{aligned}$$

where $\epsilon _{i}\in \mathcal{C}{\text{(complex)}}$ and its eigenvalues are

3 $$\begin{aligned} E_{\pm }=\frac{\epsilon _{1}+\epsilon _{2}}{2}\pm Z \end{aligned}$$

with $Z=\sqrt{\frac{(\epsilon _{1}-\epsilon _{2})^2}{4}+g^{2}}$. We denote the eigenstates corresponding to eigenvalues $E_\pm$ as $\psi _\pm$, respectively. Typically, we assume the coupling *g* to be of a real value to simplify the consideration of strong and weak interactions. Under this condition, there is a repulsion in the real part of the energy eigenvalue with a crossing in the imaginary part for $2g>|\mathrm{Im}(\epsilon _{1})-\mathrm{Im}(\epsilon _{2})|$. On the other hand, there is a repulsion in the imaginary part with a crossing in the real part for $2g<|\mathrm{Im}(\epsilon _{1})-\mathrm{Im}(\epsilon _{2})|$. The former (latter) case corresponds to the strong (weak) interaction. Especially, when $Z=0$, the eigenvalues $E_\pm$ are degenerate while the eigenfunctions $\psi _\pm$ coalesce to $|\psi _{\text{EP}}\rangle \propto |\psi _1\rangle + i |\psi _2\rangle$ (with a choice of $\pi /2$ relative phase)^41^, corresponding to an EP, where $|\psi _{1,2}\rangle$ are eigenfunctions with eigenvalues $\epsilon _{1,2}$ when $g=0$. The EP is a singular point where the transition between the strong and the weak interactions takes place^27,42^.

In the present work, we consider an elliptical dielectric two-dimensional microcavity as our open system. The closed version of this system is integrable and can be associated with a Hermitian Hamiltonian. The off-diagonal elements in Eq. (2) comes then only from the external interaction ($V_{\mathrm{SB}}G_{\mathrm{B}}^{(\mathrm{out})}V_{BS}$) in Eq. (1) in our system. The domain of eigenmodes should then be restricted to the inside of the ellipse as discussed above.

In order to study EP by two interacting modes, we need two external parameters to vary. We choose *n* the refractive index of the cavity medium and $\chi$ the deformation parameter associated with the major axis $a=R(1+\chi )$ and the minor axis $b=\frac{R}{1+\chi }$. These two parameters can be independently varied and easily controllable in actual experiments. Since functional form of the matrix elements $\epsilon _{1,2}$ and *g* on these parameters are not explicitly known, which is usually the case in many open physical systems, we rely on numerical methods to obtain the eigenvalues and their eigenmodes of electromagnetic wave confined in the dielectric cavity.

We obtained the eigenvalues and eigenmodes by solving the Helmholtz equation $\nabla ^{2}\psi +n^{2}k^{2}\psi =0$ with the boundary element method^43^ for TM electromagnetic modes in the elliptical dielectric two-dimensional cavity (in *xy* plane), where *k* is the wave number and $\psi$ is the *z*-component of the electric field.

### Shannon entropies in dielectric microcavity

The Shannon entropy for a discrete probability distribution ${\rho}_{i}$ given at *N* number of different states is defined as

4 $$\begin{aligned} S( \rho )= -\sum _{i=1}^{N}{\rho}_{i} \log {\rho}_{i}, \end{aligned}$$

with a normalized condition $\sum _{i=1}^{N}{\rho}_{i}=1$. Here, we choose the mode intensity pattern inside our cavity as the probability distribution and the *N*-mesh points for the mode intensity pattern as the *N* spatial-coordinate states of a fictitious particle in the corresponding billiard or as our *N* different states. The probability distributions are discretized at the *N*-mesh points. Note that the Shannon entropy in our case is different from the von Neumann entropy: the former is defined in terms of the probability distributions corresponding to the discretized spatial-coordinate states whereas the latter is defined by the distribution of the eigenvalue spectrum.

## Results

**Figure 1:**
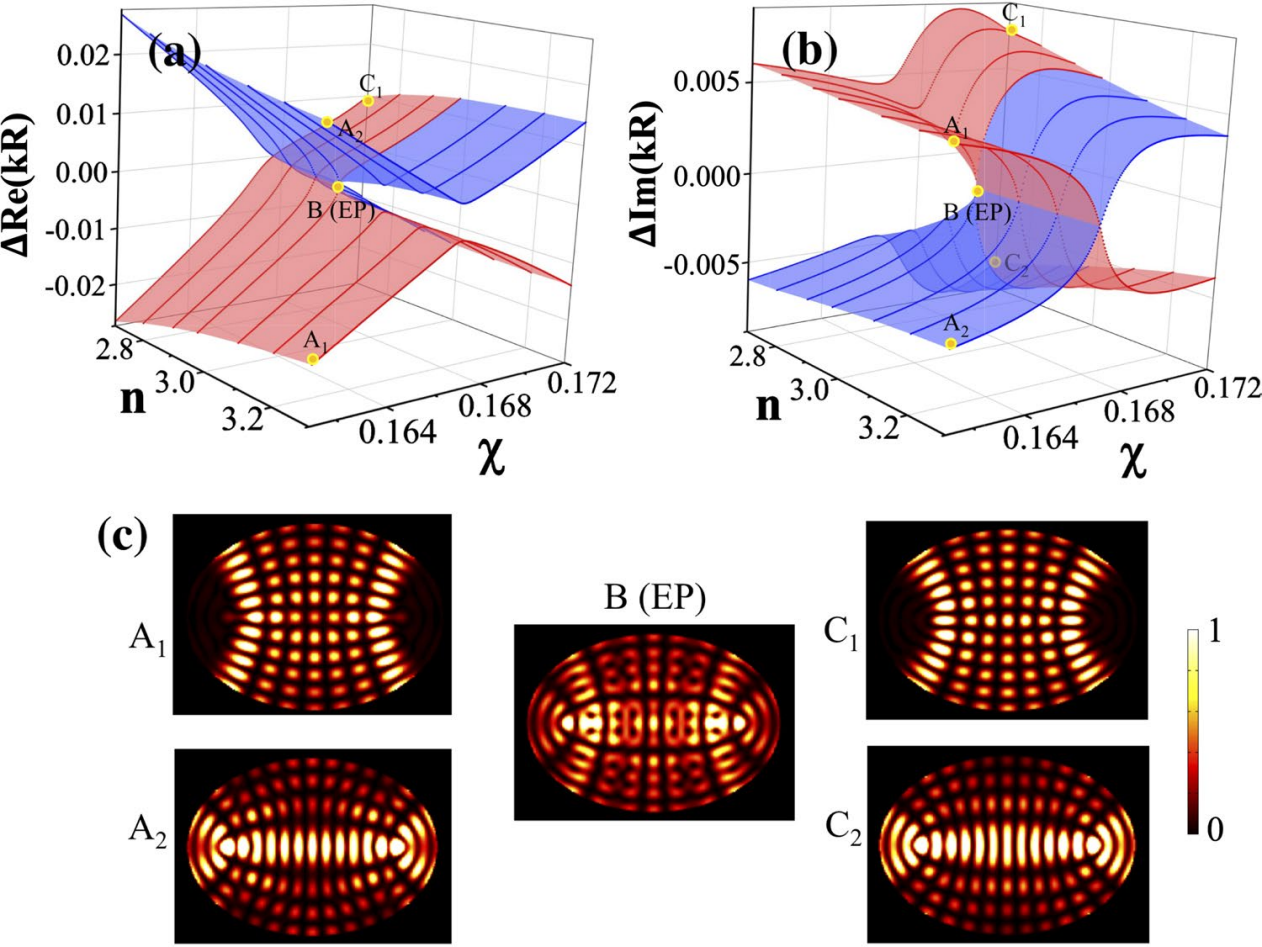
The real and imaginary parts of the eigenvalues of two interacting eigenmodes in a dielectric elliptical microcavity round an EP. (**a**) The real parts of eigenvalues ($\Delta E\pm$) in the parameter space $(n,\chi )$. They show repulsions for $n>n_{\mathrm{EP}}$ (strong interaction regime) whereas showing crossings for $n<n_{\mathrm{EP}}$ (weak interaction regime). (**b**) The imaginary parts of eigenvalues. On the contrary to the real parts, crossings occur for $n>n_{\mathrm{EP}}$ while repulsions for $n<n_{\mathrm{EP}}$. The EP is located at $(n_{\text{EP}}\simeq 2.9772, \chi _{\text{EP}}\simeq 0.16657)$. The blue (red) surface corresponds to $E_+ (E_-)$ in both (**a**) and (**b**). (**c**) The mode patterns for two interacting modes $(A_{1,2}, B, C_{1,2})$ are plotted for $(n=3.3,\chi =0.161)$, $(n \simeq n_{\text{EP}},\chi \simeq \chi _{\text{EP}})$, and $(n=2.7,\chi =0.172)$, respectively. The mode patterns are the most uniform at the EP.

We pay attention to two particular modes, which form an EP in the parameter space $s=(n,\chi )$. Their eigenvalues are depicted in Fig. 1 as a function of *n* and $\chi$ in the form of eigenvalue surfaces. In order to display the EP structure more clearly, we consider the eigenvalue offsets, $\Delta E_{\pm }=E_{\pm }-E_{\mathrm{AV}}$ with $E_{\mathrm{AV}}=\frac{E_{+}+E_{-}}{2}$, from their average values $E_{\mathrm{AV}}$ instead of the eigenvalue themselves $E_{\pm }$. The eigenvalues are presented in *kR* with *k* the complex wave number. An EP is located at $\big (n_{\text{EP}}\simeq 2.9772, \chi _{\text{EP}}\simeq 0.16657\big )$. The line $n=n_{\text{EP}}$ in Fig. 1 separates the two regimes of interactions, i.e., the strong ($n>n_{\mathrm{EP}}$) and weak ($n<n_{\mathrm{EP}}$) interactions. The mode patterns $|\psi _\pm (\mathbf{x})|^2$ of two interacting modes at three representative points (A, B and C) in the parameter space are plotted in Fig. 1c. Note that the mode pattern at the EP (B) has more uniform and chaotic probability distribution than the others (A$_{1,2}$, C$_{1,2}$).

### Shannon entropies in the vicinity of an EP

**Figure 2:**
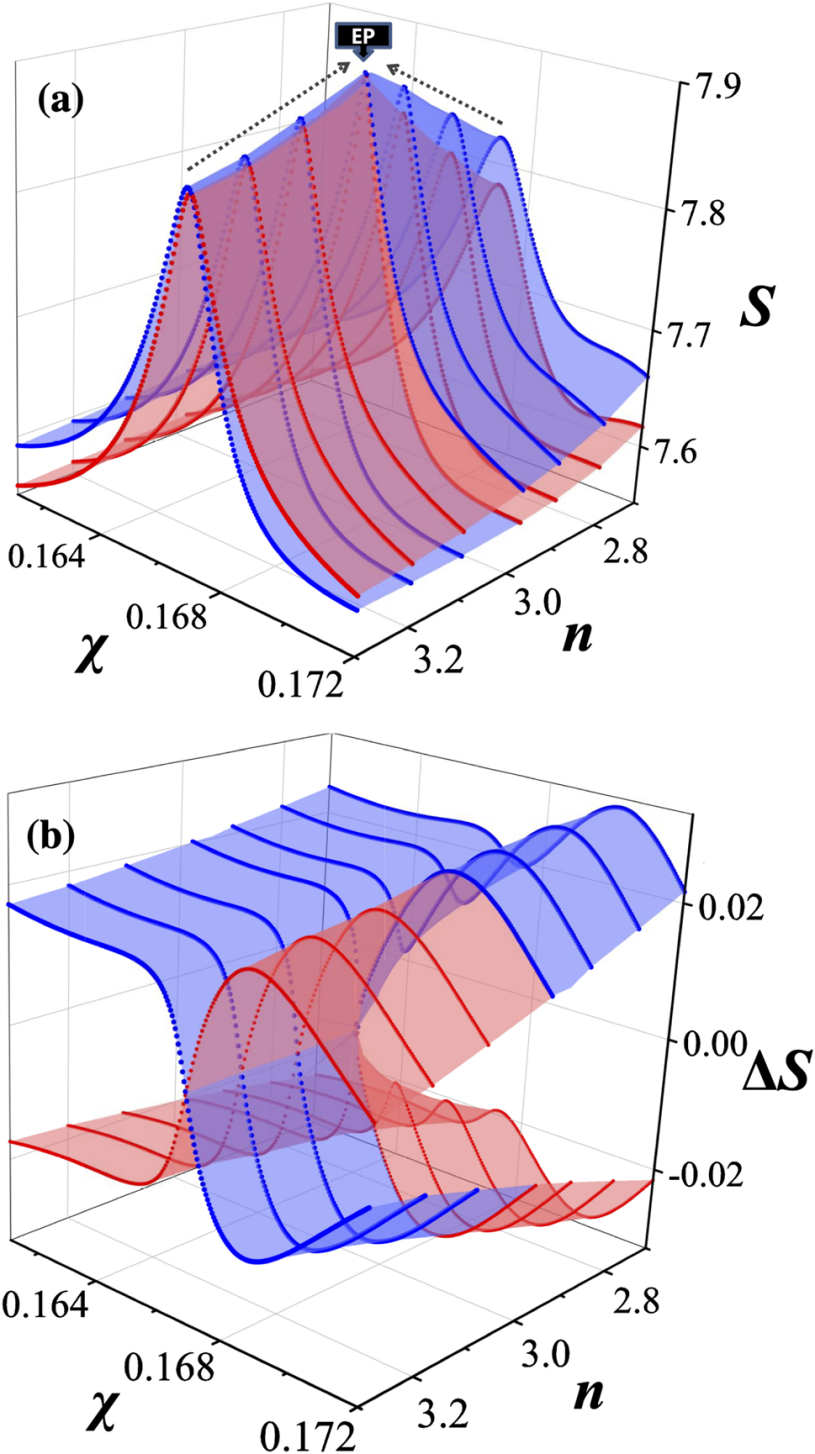
Shannon entropies of two interacting modes around the EP. (**a**) The Shannon entropy for the intensity distributions of two interacting modes in a dielectric elliptical microcavity around the EP considered in Fig. 1. The Shannon entropy is peaked at the center of interaction either in the strong or the weak interaction regime for a fixed refractive index *n*. (**b**) The Shannon entropy $\Delta S(\rho )$ with respect to a mean is obtained in the same way as in Fig. 1. The structure of $\Delta S(\rho )$ resembles that of $\Delta {\text{Im}}(kR)$ in Fig. 1b. The blue (red) surface corresponds to $\psi _+ (\psi _-)$ eigenmode in (**a**) and (**b**).

In Fig. 2, the Shannon entropies $S(\rho ; n,\chi )$ of probability density for the two interacting modes around the EP considered in Fig. 1 are plotted in the parameter space. The plots reveal two interesting features, an extreme value in the vicinity of the EP and a nontrivial topological structure around it, resembling that of the imaginary part of energy eigenvalues.

For the extreme value, we first note that the Shannon entropy is maximized near the center of interaction at a fixed refractive index *n* in both weak and strong interaction regimes. It is because the coherent superposition of eigenfunctions in either weak or strong interaction regime makes the intensity distribution more uniform. The dotted black arrows in Fig. 2a suggest that the trace of these maximum points would reach a peak of $S(\rho )\simeq 7.89$ in the vicinity of the EP. We can understand this feature by recalling that the mode function at an EP is given by $\psi _{\mathrm{EP}}\propto \psi _1+i\psi _2$, where both $\psi _1$ and $\psi _2$ can be approximately described by real wavefunctions far from the EP. As a result, the nodes of the intensity distribution occur only where two mode functions vanish simultaneously, which is extremely rare, giving rise to a distribution with a weak contrast and increased uniformity, though chaotic in pattern, and hence an increased Shannon entropy. On the other hand, when an avoided crossing occurs, we have $\psi _\pm \propto \psi _1\pm \psi _2$, which still can give a clear node structure with a strong contrast or a smaller Shannon entropy. A similar consideration can be made for mode crossing.

**Figure 3:**
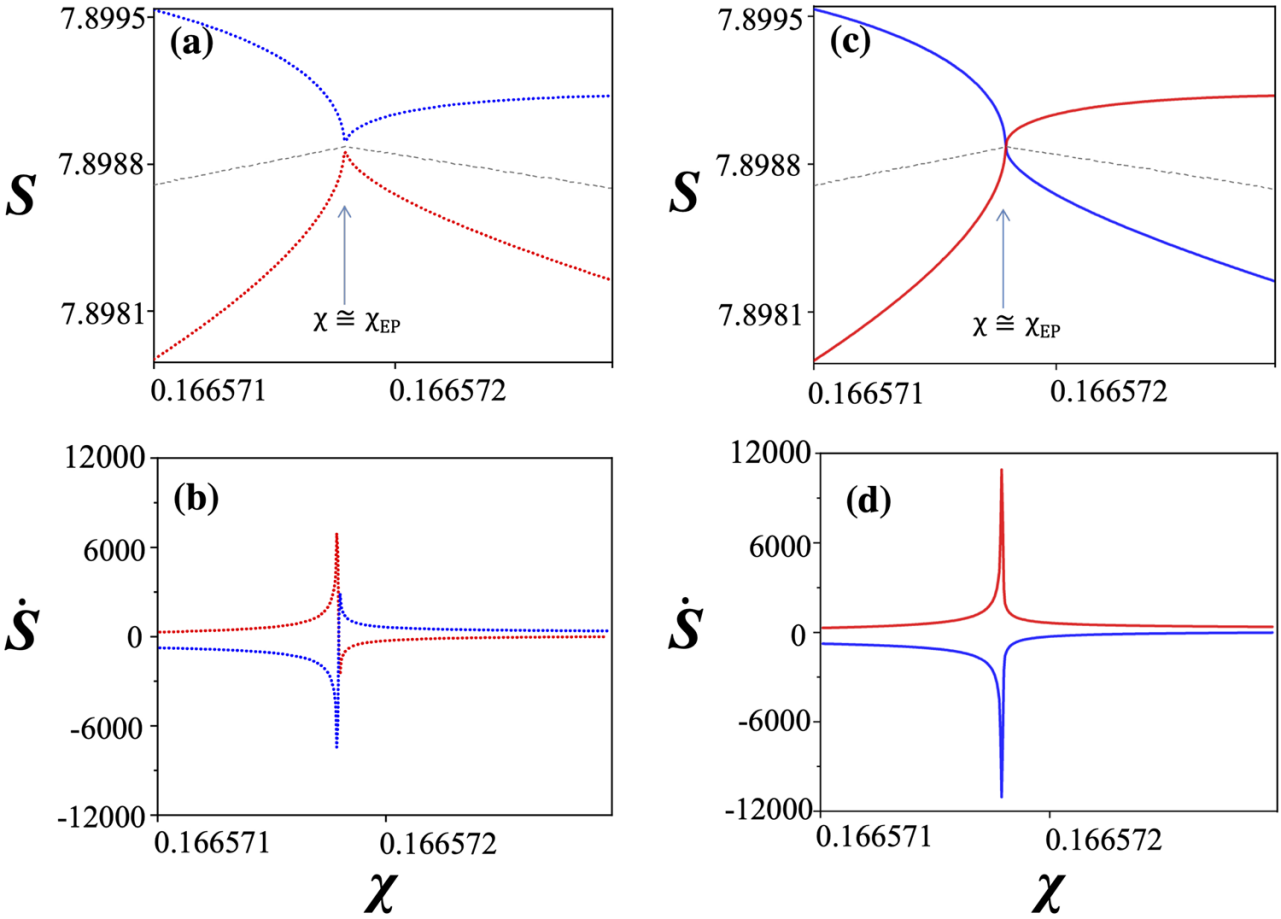
Shannon entropies and their slopes in the vicinity of the EP. (**a**) Magnified view of the Shannon entropies $S(\chi )$ and (**b**) the slopes of the Shannon entropies at $n_{-}=n_{\text{EP}}-\delta n$ with $\delta n=1\times 10^{-10}$. (**c**) Magnified view of the Shannon entropies $S(\chi )$ and (**d**) the slopes of the Shannon entropies at $n_{+}=n_{\text{EP}}+\delta n$ with $\delta n=1\times 10^{-10}$. The gray dashed line in (**a**) and (**c**) represents the average of the two Shannon entropies. The blue (red) curve corresponds to $\psi _+ (\psi _-)$ eigenmode in (**a**)–(**d**).

A close examination reveals that the maximum Shannon entropy occurs slightly off the EP. Magnified views of the Shannon entropies in the vicinity of the EP, Fig. 3a,c, show that only the mean values (dashed gray lines) of the Shannon entropies of two interacting modes show a maximum at the EP. Individual Shannon entropies either exhibit a peak and a dip structure, respectively, in the weak interaction region ($n<n_{\mathrm{EP}}$) or show rapid transitions from one branch to another in the strong interaction region ($n>n_{\mathrm{EP}}$) in the vicinity of the EP. In Fig. 3a, the Shannon entropies $S(\chi )$ are plotted as a function of $\chi$ with the refractive index fixed at $n_-=n_{\text{EP}} - \delta n$ (weak interaction regime) with $\delta n=1\times 10^{-10}$. In this case, mode crossing without exchange of mode patterns results in the repulsion of Shannon entropies with an extreme local minimum and a local maximum, respectively, at $\chi \cong \chi _{\mathrm{EP}}$. On the other hand, in Fig. 3c, the Shannon entropies $S(\chi )$ at $n_+=n_{\text{EP}} + \delta n$ (strong interaction regime), where avoided crossing with mode pattern exchange occurs, result in the crossing or the exchange of Shannon entropies at $\chi \cong \chi _{\mathrm{EP}}$. From these considerations, we can understand why the Shannon entropy surfaces in Fig. 2b resemble the imaginary part of the energy eigenvalue surfaces in Fig. 1b. Interestingly, the slopes $\dot{S}(\chi )\equiv \frac{\partial S}{\partial \chi }|_{n=n_\mp }$ of the Shannon entropies in Fig. 3b,d become divergently large as the EP is approached in both $n_{-}$ and $n_{+}$ cases. *S*(*n*) and $\dot{S}(n)$ at $\chi _{\pm }=\chi _{\mathrm{EP}}\pm \delta \chi$ with $\delta \chi =10^{-10}$ behave in a similar way.

## Discussion

### Diverging slopes of Shannon entropies very near an EP

In order to understand the behaviors of the Shannon entropy and its divergent slope very near the EP, let us consider the Shannon entropy as a function of small perturbation $\epsilon$ in the vicinity of the EP by using the Newton-Puiseux series^44^. The eigenvalue equations in the vicinity of an EP can be written as

5 $$\begin{aligned}&(H_{\mathrm{EP}}+\epsilon H_{1}+\cdots )\left( |\psi _{\mathrm{EP}}\rangle +\epsilon ^{\frac{1}{2}}|{\psi }_{1}\rangle +\cdots \right) \nonumber \\&=(\lambda _{\mathrm{EP}}+\epsilon ^{\frac{1}{2}}{\lambda }_{1}+\cdots )\left( |\psi _{\mathrm{EP}}\rangle +\epsilon ^{\frac{1}{2}}|{\psi }_{1}\rangle +\cdots \right) . \end{aligned}$$

Using this expansion we can express the probability density $\rho _\pm (\mathbf{x},\epsilon )=|\psi _\pm (\mathbf{x},\epsilon )|^{2}$ as

6 $$\begin{aligned} \rho _\pm (\mathbf{x},\epsilon )&\simeq |\psi _{\mathrm{EP}}(\mathbf{x})|^2 + \epsilon |c|^2|J_{\mathrm{EP}}(\mathbf{x})|^2 \nonumber \\&\;\;\; \pm 2\sqrt{\epsilon }\mathrm{Re}\left[ c\psi _{\mathrm{EP}}^*(\mathbf{x})J_{\mathrm{EP}}(\mathbf{x})\right] , \end{aligned}$$

where $c=\sqrt{\langle \phi _{\mathrm{EP}}|H_{1}|\psi _{\mathrm{EP}}\rangle }$ with $\langle \phi _{\mathrm{EP}}|$ the adjoint of $|\psi _{\mathrm{EP}}\rangle$ and $J_{\mathrm{EP}}(\mathbf{x})$ is the Jordan vector at the EP (see “Methods”). Note that the probability density is given by $\rho (\mathbf{x})\equiv |\psi (\mathbf{x})|^2$ for the electromagnetic eigenmode $\psi (\mathbf{x})$ of a dielectric microcavity. Consequently, the Shannon entropy near the EP can be expanded to the lowest order of $\epsilon$ as

7 $$\begin{aligned} S_\pm (\epsilon )&\simeq S_{\mathrm{EP}} \mp 2\sqrt{\epsilon } \sum _{i=1}^{N}\mathrm{Re}\left[ c\psi _{\mathrm{EP}}^*(\mathbf{x}_{i})J_{\mathrm{EP}}(\mathbf{x}_{i})\right] \nonumber \\ & \quad \times \left\{ \log \left[ |\psi _{\mathrm{EP}}(\mathbf{x}_i)|^2\right] +1\right\} , \end{aligned}$$

and its derivative as

8 $$\begin{aligned} \frac{d S_\pm (\epsilon )}{d \epsilon }&\simeq \mp \frac{1}{\sqrt{\epsilon }} \sum _{i=1}^{N}\mathrm{Re}\left[ c\psi _{\mathrm{EP}}^*(\mathbf{x}_{i})J_{\mathrm{EP}}(\mathbf{x}_{i})\right] \nonumber \\ & \quad \times \left\{ \log \left[ |\psi _{\mathrm{EP}}(\mathbf{x}_i)|^2\right] +1\right\} , \end{aligned}$$

and therefore $\frac{d S_\pm (\epsilon )}{d \epsilon }\rightarrow \mp \infty$ as $\epsilon \rightarrow 0$. Equations (7) and (8) explain the branching of the Shannon entropies at the EP with divergent slopes as shown in Fig. 3. The fact that the slopes diverge at the EP indicates the Shannon entropy would change rapidly under perturbations in the vicinity of the EP. These property can be used to build a sensor operating at an EP by monitoring the eigenmode distribution.

**Figure 4:**
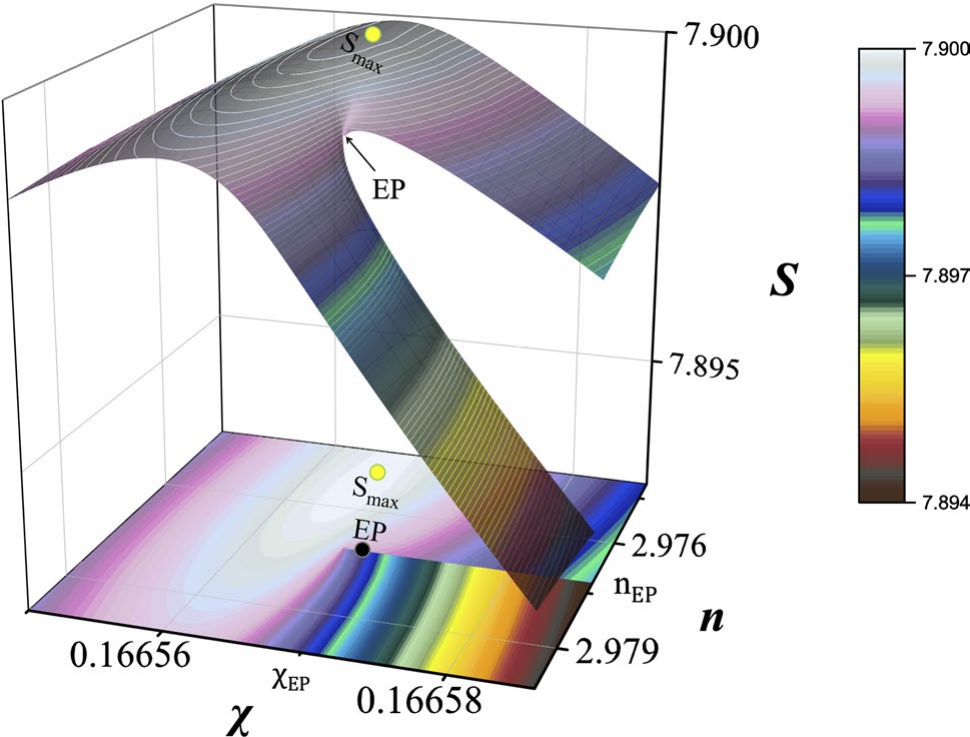
Global maximum of Shannon entropies. The global maximum $S_{\text{max}}\cong 7.89996$ occurs for $\psi _+$ mode of eigenvalue $E_+$, located at $\chi \cong \chi _{\mathrm{EP}}-0.00005\simeq 0.9997 \chi _{\mathrm{EP}}$ and $n\cong n_{\mathrm{EP}}-0.002\simeq 0.9993 n_{\mathrm{EP}}$.

It is noteworthy that the separation (repelling) of two Shannon entropies is larger for $\chi < \chi _{\mathrm{EP}}$ (also for $n < n_{\mathrm{EP}}$ although not shown) than for $\chi > \chi _{\mathrm{EP}}$ (or $n >n_{\mathrm{EP}}$) in Fig. 3a,c. This is due to the fact that the strong (weak) interactions between two modes induce the strong (weak) mixture of their eigenfunctions, resulting in the more (less) similar Shannon entropies. Consequently, the global maximum $(S_{\text{max}}\cong 7.89996)$ of the Shannon entropy occurs toward the doubly weak interaction region specified by $\chi <\chi _{\mathrm{EP}}$ and $n < n_{\mathrm{EP}}$ as shown in Fig. 4. The displacement of the global maximum of Shannon entropy from the EP is extremely small, in the order of $\Delta \chi /\chi _{\mathrm{EP}}\simeq 3\times 10^{-4}$ and $\Delta n/n_{\mathrm{EP}}\simeq 7\times 10^{-4}$. The global maximum of the average of two Shannon entropies occurs exactly at the EP as already discussed in Fig. 3.

These two observations support our conjecture that the mode distribution at the EP might contain the largest information contents or exhibit the most uniformly complex spatial patterns, roughly speaking. The statement is true for the average Shannon entropy and approximately true for the global maximum. Based on this confirmation, one may then legitimately ask the relation of the most uniformly complex mode pattern at the EP and the increased vacuum fluctuations occurring in the EP mode. The answers to such investigation, which is beyond the scope of the present work and is thus left for a future study, would then shed light on the current conflicting experimental observation of an increased linewidth^29^ to the classical theory predicting no such enhancement^30^.

### Topological structure of the Shannon entropy in the vicinity of an EP

**Figure 5:**
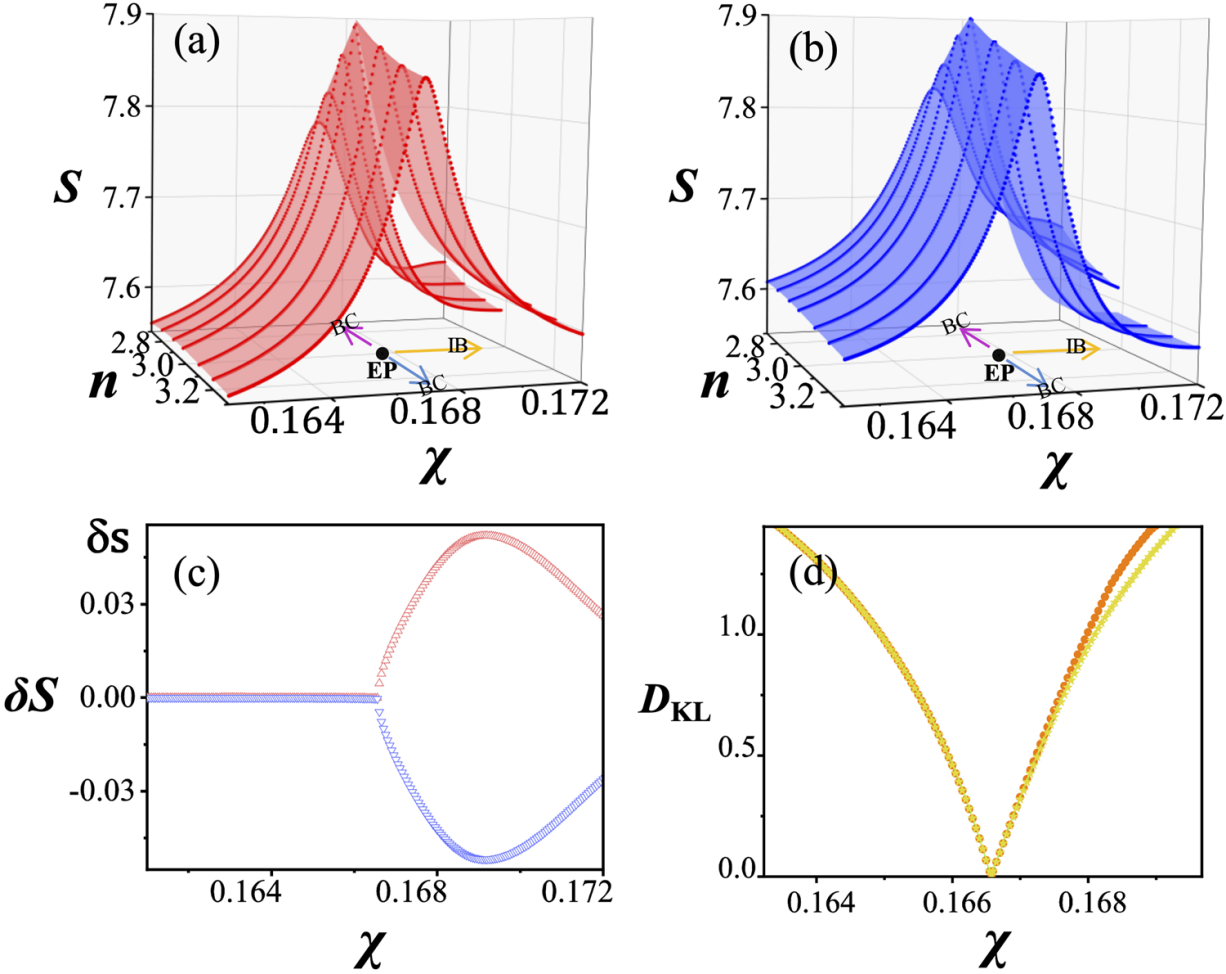
Topological structure of Shannon entropies around the EP. (**a**) The Shannon entropy for $\psi _-$ state and (**b**) for $\psi _+$ state drawn individually. The discontinuity appears at the line $n\simeq n_{\text{EP}}$ in both cases. The EP as a branch point, the branch cut (BC)—blue (red) arrow for the real (imaginary) part of the eigenvalue—and the interaction branch (IB) are shown on the base planes. (**c**) The difference $\delta S(\rho )$ between the Shannon entropies at $n=n_{-}$ and $n=n_{+}$ for $\psi _-$ (red dots) and $\psi _+$ (blue dots) states. (**d**) The KL divergence ${D}_{\mathrm{KL}}$ or the relative entropy at $n=n_+$ (orange dots) and at $n=n_-$ (yellow dots) for two interacting modes.

For the nontrivial topological structure of the Shannon entropy seen in Fig. 2a,b, we note that the two cyclic variations are required for the Shannon entropy values to return to the original values on the Shannon entropy surfaces, just like the imaginary part of the complex energy surfaces shown in Fig. 1b. The surface discontinuity is exhibited along the line $n\simeq n_{\mathrm{EP}}$ for $\chi >\chi _{\mathrm{EP}}$—let us call this line the interaction branch (IB)—for both modes in Fig. 5a,b. This feature can be quantified by $\delta S(\rho )=S(\rho ;n_{-},\chi )-S(\rho ;n_{+},\chi )$ as shown in Fig. 5c, where $\delta S(\rho )$ remains almost zero for $\chi < \chi _{\mathrm{EP}}$ whereas it increases significantly for $\chi > \chi _{\mathrm{EP}}$. The discontinuity of the Shannon entropy surfaces across the IB is directly related to the exchange of the Shannon entropy as well as the mode pattern exchange.

This exchange property can be further quantified by introducing the relative entropy or the Kullback-Leibler (KL) divergence, which is a measure of the distance between two probability distributions of a random variable^45^. The KL divergence from *Q* to *P*, $D_{\text{KL}}\big (P\parallel Q\big )$, is defined by

9 $$\begin{aligned} D_{\text{KL}}(P\parallel Q)= -\sum _{i=1}^{N}P(\mathbf{x}_{i})\log \frac{Q(\mathbf{x}_{i})}{P(\mathbf{x}_{i})}. \end{aligned}$$

The KL divergence for the two interacting modes along the $n_\pm$ lines, respectively, is plotted in Fig. 5d. It is seen that the KL divergences are almost the same when $\chi <\chi _{\mathrm{EP}}$ and they become zero at the EP. However, their difference $\Delta D^{w,s}_{\text{KL}}$ becomes larger across the interaction branch when $\chi >\chi _{\text{EP}}$. These results are consistent with the fact that the mode patterns as well as the Shannon entropies in the weak interaction regime are not exchanged whereas those in the strong interaction regime are exchanged.

## Methods

### Series expansion of Shannon entropy and its derivative near an EP

We performed the series expansion of the eigenvectors and eigenvalues near an EP following the method described in Ref.^44^. At an EP, two eigenvectors coalesce to one eigenvector $|\psi _{\mathrm{EP}}\rangle$ with an eigenvalue $\lambda _{\mathrm{EP}}$.

10 $$\begin{aligned} (H_{\mathrm{EP}}-\lambda _{\mathrm{EP}})|\psi _{\mathrm{EP}}\rangle =0 \end{aligned}$$

Its adjoint satisfies

11 $$\begin{aligned} \langle \phi _{\mathrm{EP}}|(H_{\mathrm{EP}}-\lambda _{\mathrm{EP}})=0. \end{aligned}$$

For completeness at the EP, we can define the so-called Jordan vector $|J_{\mathrm{EP}}\rangle$ with the relation

12 $$\begin{aligned} (H_{\mathrm{EP}}-\lambda _{\mathrm{EP}})|J_{\mathrm{EP}}\rangle =|\psi _{\mathrm{EP}}\rangle . \end{aligned}$$

and its adjoint by

13 $$\begin{aligned} \langle I_{\mathrm{EP}}|(H_{\mathrm{EP}}-\lambda _{\mathrm{EP}})=\langle \phi _{\mathrm{EP}}|. \end{aligned}$$

The Jordan vector and its adjoint are subject to the normalization conditions $\left\langle \phi _{\mathrm{EP}}|J_{\mathrm{EP}}\right\rangle =\left\langle I_{\mathrm{EP}}|\psi _{\mathrm{EP}}\right\rangle =1$ and $\left\langle I_{\mathrm{EP}}|J_{\mathrm{EP}}\right\rangle =0$.

In order to obtain the expressions for the eigenvalues and eigenvectors near the EP, let us consider the Taylor expansion of the Hamiltonian in terms of a small perturbative parameter $\epsilon \ll 1$ near the EP.

14 $$\begin{aligned} H=H_{\mathrm{EP}}+\epsilon H_1+\cdots . \end{aligned}$$

Note that the eigenvalues and eigenvectors in the vicinity of EP can be expanded in fractional powers of $\epsilon$ (Newton-Puiseux series). Hence, the eigenvalue equations in the vicinity of EP can be written as

15 $$\begin{aligned}&(H_{\mathrm{EP}}+\epsilon H_{1}+\cdots )\left( |\psi _{\mathrm{EP}}\rangle +\epsilon ^{\frac{1}{2}}|{\psi _{1}}\rangle +\cdots \right) \nonumber \\&\quad =(\lambda _{\mathrm{EP}}+\epsilon ^{\frac{1}{2}}{\lambda _{1}}+\cdots )\left( |\psi _{\mathrm{EP}}\rangle +\epsilon ^{\frac{1}{2}}|{\psi _{1}}\rangle +\cdots \right) . \end{aligned}$$

Equating terms corresponding to different powers of $\epsilon$, we obtain the following relations for the leading orders.

16 $$\begin{aligned} H_{\mathrm{EP}}|\psi _{\mathrm{EP}}\rangle =&\lambda _{\mathrm{EP}}|\psi _{\mathrm{EP}}\rangle , \end{aligned}$$

17 $$\begin{aligned} H_{\mathrm{EP}}|\psi _{1}\rangle =&(\lambda _{\mathrm{EP}}|{\psi _{1}}\rangle +\lambda _{1}|\psi _{\mathrm{EP}}\rangle ), \end{aligned}$$

18 $$\begin{aligned} (H_{\mathrm{EP}}|\psi _{2}\rangle +H_{1}|\psi _{\mathrm{EP}}\rangle )=&(\lambda _{\mathrm{EP}}|\psi _{2}\rangle +\lambda _{1}|\psi _{1}\rangle +\lambda _{2}|\psi _{\mathrm{EP}}\rangle ). \end{aligned}$$

Comparing the Eq. (12) with Eq. (17) yields the result:

19 $$\begin{aligned} |\psi _{1}\rangle =\lambda _{1}|J_{\mathrm{EP}}\rangle . \end{aligned}$$

In order to find the $\lambda _{1}$, let us rewrite Eq. (18) using Eq. (19):

20 $$\begin{aligned}&(H_{\mathrm{EP}}-\lambda _{\mathrm{EP}})|\psi _{2}\rangle \nonumber \\&\quad =\lambda ^{2}_{1}|{J}_{\mathrm{EP}}\rangle +\lambda _{2}|\psi _{\mathrm{EP}}\rangle -H_{1}|\psi _{\mathrm{EP}}\rangle . \end{aligned}$$

With Eq. (20) multiplied by $\langle \phi _{\mathrm{EP}}|$ from the left, we get $\langle \phi _{\mathrm{EP}}|[\lambda ^{2}_{1}|\psi ^{J}_{\mathrm{EP}}\rangle +\lambda _{2}|\psi _{\mathrm{EP}}\rangle -H_{1}|\psi _{\mathrm{EP}}\rangle ]=0$ since $\langle \phi _{\mathrm{EP}}|(H_{\mathrm{EP}}-\lambda _{\mathrm{EP}})=0$. Using the normalization condition for the Jordan vector, we find

21 $$\begin{aligned} \lambda ^{2}_{1}=\langle \phi _{\mathrm{EP}}|H_{1}|\psi _{\mathrm{EP}}\rangle . \end{aligned}$$

As a result, the two values of $\lambda _{1}=\pm c$ with $c=\sqrt{\langle \phi _{\mathrm{EP}}|H_{1}|\psi _{\mathrm{EP}}\rangle }$ determine the leading terms in expansions. So the eigenvalues are

22 $$\begin{aligned} \lambda _{\pm }(\epsilon )\simeq \lambda _{\mathrm{EP}}\pm c\epsilon ^{\frac{1}{2}} +O(\epsilon ) \end{aligned}$$

and the eigenvectors are

23 $$\begin{aligned} |\psi _{\pm }(\epsilon )\rangle \simeq |\psi _{\mathrm{EP}}\rangle \pm c\epsilon ^{\frac{1}{2}}|J_{\mathrm{EP}}\rangle +O(\epsilon ). \end{aligned}$$

Now let us calculate the Shannon entropy and its derivative. Shannon entropy in our case is defined as

24 $$\begin{aligned} S=-\sum _{i=1}^N \rho (\mathbf{x}_i) \log \rho (\mathbf{x}_i) \end{aligned}$$

for the probability density $\rho (\mathbf{x})\equiv |\psi (\mathbf{x})|^2$ of a classical eigenmode $\psi (\mathbf{x})$ of a two-dimensional dielectric microcavity. Near an EP, the probability density $\rho _\pm (\mathbf{x}_{i},\epsilon )=|\psi _\pm (\mathbf{x}_{i},\epsilon )|^{2}$ are given by

25 $$\begin{aligned} \rho _\pm (\mathbf{x},\epsilon )&\simeq |\psi _{\mathrm{EP}}(\mathbf{x})|^2 \pm 2\sqrt{\epsilon }\mathrm{Re}\left[ c\psi _{\mathrm{EP}}^*(\mathbf{x})J_{\mathrm{EP}}(\mathbf{x})\right] \end{aligned}$$

up to the lowest order of $\epsilon$. Then, the Shannon entropy in the vicinity of the EP can be written as

26 $$\begin{aligned} S_{\pm }(\epsilon )&= -\sum _{i=1}^{N}\rho _{\pm }(\mathbf{x}_i,\epsilon ) \log \rho _{\pm }(\mathbf{x}_i,\epsilon )\nonumber \\&\simeq -\sum _{i=1}^{N}\left\{ |\psi _{\mathrm{EP}}(\mathbf{x}_{i})|^2 \pm 2\sqrt{\epsilon }\mathrm{Re} \left[ c\psi _{\mathrm{EP}}^*(\mathbf{x}_{i})J_{\mathrm{EP}}(\mathbf{x}_{i})\right] \right\} \nonumber \\ & \quad \times {\left\{ \log \left[ |\psi _{\mathrm{EP}}(\mathbf{x}_{i})|^2\right] \pm \frac{2\sqrt{\epsilon }\mathrm{Re} \left[ c\psi _{\mathrm{EP}}^*(\mathbf{x}_{i})J_{\mathrm{EP}}(\mathbf{x}_{i})\right] }{|\psi _{\mathrm{EP}}(\mathbf{x}_{i})|^2}\right\} } \nonumber \\&= S_{\mathrm{EP}} \mp 2\sqrt{\epsilon } W_1, \end{aligned}$$

where $S_{\mathrm{EP}} \equiv - \sum _{i=1}^N |\psi _{\mathrm{EP}}(\mathbf{x}_{i})|^2 \log \left[ |\psi _{\mathrm{EP}}(\mathbf{x}_{i})|^2\right]$ and $W_1\equiv \mathrm{Re}\sum _{i=1}^{N} \left[ c\psi _{\mathrm{EP}}^*(\mathbf{x}_{i})J_{\mathrm{EP}}(\mathbf{x}_{i})\right] \left\{ \log \left[ |\psi _{\mathrm{EP}}(\mathbf{x}_{i})|^2\right] +1\right\}$. Hence, the dominant term $\Delta S(\epsilon )$ for the separation of the Shannon entropy at EP is given by

27 $$\begin{aligned} \Delta S(\epsilon )\equiv S_\pm (\epsilon )-S_{\mathrm{EP}} \approx \mp 2\sqrt{\epsilon } W_1 \end{aligned}$$

The derivative of the Shannon entropy with respect to $\epsilon$ is given by

28 $$\begin{aligned} \frac{d S_\pm (\epsilon )}{d \epsilon }=-\sum _{i=1}^{N} \left[ \log (\rho _\pm (\mathbf{x}_{i},\epsilon )) + 1\right] \frac{\partial \rho _\pm (\mathbf{x}_{i},\epsilon )}{\partial \epsilon }. \end{aligned}$$

Using

29 $$\begin{aligned} \frac{\partial \rho _\pm (\mathbf{x}_{i},\epsilon )}{\partial \epsilon }\simeq \pm \frac{1}{\sqrt{\epsilon }}\mathrm{Re}\left[ c\psi _{\mathrm{EP}}^*(\mathbf{x}_{i})J_{\mathrm{EP}}(\mathbf{x}_{i})\right] \end{aligned}$$

as $\epsilon \rightarrow 0$, we obtain

30 $$\begin{aligned} \frac{d S_\pm (\epsilon )}{d \epsilon }\simeq \mp {\frac{W_1}{\sqrt{\epsilon }}} \end{aligned}$$

As a result, $\frac{d S_\pm (\epsilon )}{d \epsilon }\rightarrow \mp \infty$ as $\epsilon \rightarrow 0$. Figure 3 shows these features clearly.
